# Serum HBV surface antigen positivity is associated with low prevalence of metabolic syndrome: A meta-analysis

**DOI:** 10.1371/journal.pone.0177713

**Published:** 2017-05-15

**Authors:** Yuanyuan Li, Ying Zhao, Jianping Wu

**Affiliations:** Department of Laboratory Medicine, The First Affiliated Hospital, College of Medicine, Zhejiang University, Hangzhou, China; Universita degli Studi di Pisa, ITALY

## Abstract

**Background and aim:**

As there is conflicting evidence for the relationship between hepatitis B virus surface antigen (HBsAg) positivity and the prevalence of metabolic syndrome (MetS), we performed a meta-analysis to investigate whether HBsAg positivity affects the incidence of MetS.

**Methods:**

Observational studies on the relationship between HBsAg positivity and MetS were obtained from PubMed, Web of Science, and the Cochrane Library in April 2016. The pooled odds ratios (ORs) of MetS and its components (central obesity, increased fasting glucose, increased blood pressure, dyslipidemia) for subjects with or without HBsAg positivity were synthesized. The standardized mean difference of MetS components between HBsAg-positive participants and healthy controls was calculated. Heterogeneity was explored with subgroup analysis and sensitivity analysis. Publication bias was detected using Egger’s test and Begg’s test.

**Results:**

Thirty studies were eligible for meta-analysis. The MetS OR for HBsAg-positive participants was significantly decreased compared with the controls [OR = 0.80, 95% confidence interval (CI), 0.70–0.90]. The negative effect of HBsAg positivity on elevated triglycerides (OR = 0.62, 95% CI, 0.59–0.64) was strong, while that for increased fasting blood glucose was weak (OR = 0.94, 95% CI, 0.90–0.98). The pooled ORs of central obesity (OR = 0.97, 95% CI, 0.91–1.04), reduced high-density lipoprotein cholesterol (OR = 0.98, 95% CI, 0.83–1.14), and elevated blood pressure (OR = 1.00, 95% CI, 0.80–1.25) for HBsAg-positive participants were all not significantly different compared with the controls. No publication bias was detected.

**Conclusions:**

Serum HBsAg positivity is inversely associated with the prevalence of MetS. Among the five components of MetS, elevated triglycerides had the strongest inverse relationship with HBsAg positivity.

## Introduction

Chronic hepatitis B virus (HBV) infection remains a globally challenging problem, as it can lead to chronic active hepatitis, liver cirrhosis, and hepatocellular carcinoma [1, 2]. Metabolic syndrome (MetS), characterized by a cluster of metabolic abnormalities including central obesity, increased fasting blood glucose (FBG), increased blood pressure (BP), and dyslipidemia, is another issue of global concern. MetS is a confirmed risk factor for type 2 diabetes mellitus and atherosclerotic cardiovascular disease [3], and its prevalence has grown rapidly over the past two decades [4].

The liver plays an undeniably important role in lipid and glucose metabolism. MetS involves dyslipidemia and glucose abnormalities. Dyslipidemia is associated with the development of obesity and hypertension, which are also components of MetS. Additionally, nonalcoholic steatohepatitis is considered the hepatic manifestation of MetS [5, 6], and MetS and nonalcoholic steatohepatitis are mutual promoters [7, 8]. Overall, MetS is related to the liver in some way. The hepatitis virus damages liver function; does it also disrupt the metabolism of lipids and glucose in the liver? Subsequently, does it affect the incidence of MetS?

HBV and hepatitis C virus (HCV) are two common types of hepatitis virus that share some similarities. Chronic HCV infection contributes to MetS, as it induced insulin resistance in a genotype-dependent model [9]. However, the relationship between HBV and MetS in the literature, including large population-based surveys, remains inconclusive. HBV surface antigen (HBsAg) positivity and HBV infection are not synonymous, e.g., there can be occult HBV infection with HBsAg-negative status. Even so, HBsAg positivity is closely related to various HBV infection statuses (HBV carrier, chronic active hepatitis, liver cirrhosis). Consequently, HBsAg is usually an indicator of HBV infection. Some studies [10–14] concluded that HBsAg seropositivity is a protective factor against MetS, while others [15–17] have found no association between HBsAg positivity and MetS. These conflicting evidences render a systematic assessment necessary. Unfortunately, the relevant systematic analysis has not been performed. Therefore, we performed this meta-analysis to investigate whether HBsAg seropositivity affects the incidence of MetS and whether HBsAg positivity is related to the components of MetS (central obesity, increased FBG, increased BP, dyslipidemia).

## Materials and methods

### Search strategy

This meta-analysis was performed according to a proposal for reporting meta-analysis of observational studies [18]. We searched the following databases without time limitations: PubMed, Web of Science, the Cochrane Library. The search strategy for identifying all relevant literature used the following keywords: hepatitis B, metabolic syndrome, hypertension, hyperglycemia, hypertriglyceridemia, dyslipidemia (see S1 Text). The literature search was updated in April 2016.

### Study selection

Studies were deemed eligible if they met the following criteria: (1) investigated the association between HBsAg positivity and MetS (including components of MetS: central obesity; increased triglyceride [TG]; reduced high-density lipoprotein cholesterol [HDL-C]; increased BP; increased FBG). HBV infection was defined as HBsAg seropositivity; (2) used healthy subjects as the control group; (3) included >30 subjects with HBsAg positivity; otherwise, a study was excluded for low statistical power and poor reliability. Exclusion criteria were studies on co-infection, such as human immunodeficiency virus and HBV co-infection, liver cirrhosis, hepatocarcinoma, following antiviral therapy, pregnant or pediatric populations.

### Methodological quality assessment and data extraction

Two authors (L.Y.Y. and Z.Y.) independently assessed the quality of eligible studies. The Newcastle-Ottawa Scale criteria [19] were recommended by the Cochrane Collaboration for assessing the quality of nonrandomized studies in a meta-analysis. As it was suitable for case-control and cohort studies, we modified it for cross-sectional studies (Table 1). An additional explanation was needed for Q4, which involved the definition of MetS and its components. MetS was defined as the presence of three or more of the following items [4, 20, 21]: (1) elevated waist circumference (WC) (population- and country-specific definitions); (2) elevated TG (≥150 mg/dL) or therapy; (3) reduced HDL-C (men, <40 mg/dL; women, <50 mg/dL) or therapy; (4) elevated BP (systolic ≥ 130 mmHg and/or diastolic ≥ 85 mmHg) or therapy; (5) elevated FBG or therapy. Elevated FBG was defined slightly differently (≥100 mg/dL [20] and ≥110 mg/dL [21]). Both were allowable in this meta-analysis, and further subgroup analysis was performed. The checklist of Q4 was that “The MetS and its components were defined accurately”. Here, the accurate definition of MetS must meet the above criteria. The accurate definition of a MetS component must match the corresponding item of the MetS component. For example, one study focused only on the relationship between HBsAg positivity and TG (one component of MetS), and the cutoff value for calculating the odds ratio (OR) for elevated TG was identical with the item of MetS (TG ≥ 150 mg/dL). This study was also awarded one star for Q4. Discrepancies during methodological quality assessment were resolved by consensus agreement.

**Table 1:** Checklist of methodological quality assessment.

Code	Checklist
Q1	The participants were recruited from general population, and were not from hospital;
Q2	The subjects with HBsAg positivity and controls were from the same community;
Q3	The experimental group was composed of subjects with HBsAg positivity;
Q4^†^	The MetS and its components were defined accurately;
Q5	The same detection method was applied to subjects with HBsAg positivity and controls;
Q6	The same diagnostic criteria were applied to define MetS and its components for subjects with HBsAg positivity and controls;
Q7	The studies list inclusion and exclusion criteria, and patients with hepatitis C virus infection should be excluded at least;
Q8	The studies which were included to calculate combined standardized mean difference were matched for age and sex at least. The studies which were included to calculate combined odds ratio were adjusted for age and sex at least;
Q9	The lifestyle (alcohol and smoking at least) should be considered. The confounding factors from lifestyle were not significantly different between subjects with HBsAg positivity and controls; or they were adjusted in calculating odds ratio.

MetS, metabolic syndrome; HBsAg, hepatitis B surface antigen;

^†^, MetS was defined as the presence of three or more of the following items: (1) elevated waist circumference (population- and country-specific definitions); (2) elevated triglycerides (≥150 mg/dL) or therapy; (3) reduced high-density lipoprotein cholesterol (<40 mg/dL in men; <50 mg/dL in women) or therapy; (4) elevated blood pressure (systolic ≥ 130 mmHg and/or diastolic ≥ 85 mm Hg) or therapy; (5) elevated fasting blood glucose (≥100 mg/dL or ≥110 mg/dL) or therapy. The accurate definition of MetS must meet the above criteria. The accurate definition of a MetS component must match the corresponding item of the MetS component. For example, one study focused only on the relationship between HBsAg positivity and TG (one component of MetS), and the cutoff value for calculating the OR for elevated TG was identical with the item of MetS (TG ≥ 150 mg/dL). This study was also awarded one star for Q4.

For continuous variables, the mean and standard deviation (SD) of WC, body mass index (BMI), TG, HDL-C, FBG, systolic BP, and diastolic BP for HBsAg positive subjects and the controls were extracted. For categorical variables, the adjusted OR was extracted; otherwise, the crude data were extracted to calculate the OR. In addition, the datasheet included the publication year, region, study design, source of subjects, sample size, mean age, gender distribution, and diagnostic criteria of MetS.

### Statistical analysis

The standardized mean difference (SMD) of WC, BMI, TG, HDL-C, FBG, systolic BP, and diastolic BP between the HBsAg-positive group and controls was calculated. Then, the pooled SMD and associated 95% confidence intervals (CI) were obtained from a DerSimonian and Laird random effects model [22]. More importantly, pooled OR was selected to assess the relationship between HBsAg positivity and MetS. Heterogeneity between eligible studies was evaluated by the I^2^ test. The degree of heterogeneity was classified to three levels (minimal, I^2^ < 25%; moderate, 25% ≤ I^2^ < 50%; substantial, I^2^ ≥ 50%) [23]. If no significant heterogeneity was detected (*P* > 0.05 and I^2^ < 50%), the fixed effect model was used to calculate the pooled OR and 95% CI. Otherwise, the random effect model was used. To investigate the source of heterogeneity, subgroup analysis and sensitivity analysis was performed according to the factors related to quality assessment. Publication bias was assessed with Egger’s test [24] and Begg’s test [25] (significance at *P* < 0.05). Statistical analyses were conducted with Review Manager 5.3 (The Cochrane Collaboration) and STATA 11.0 (Stata Corp., College Station, TX, USA).

## Results

### Study characteristics

**Fig 1:**
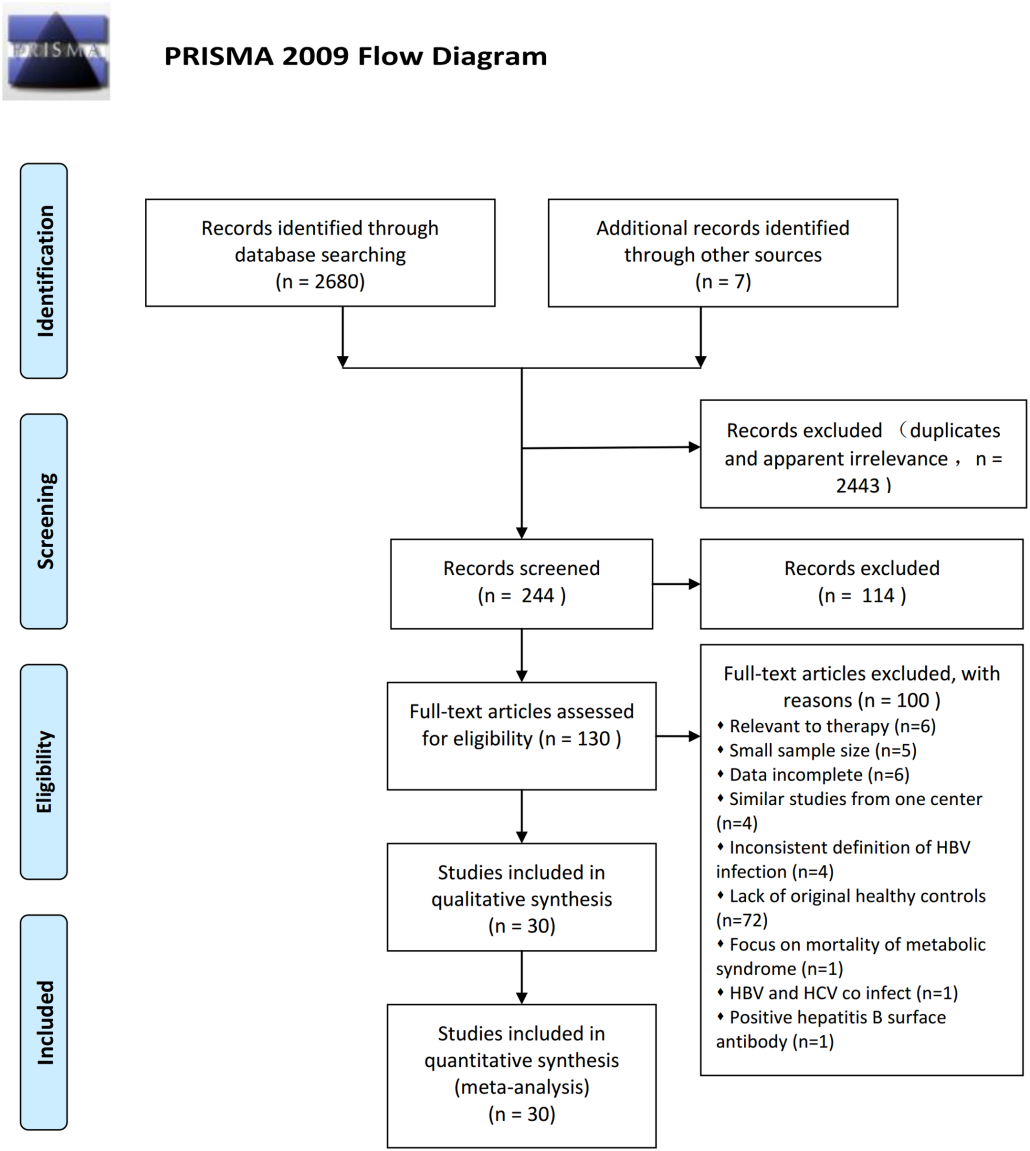
Flow diagram of screened, excluded, and analyzed literature.

We retrieved 2687 studies using the described search strategies. We excluded 2657 studies in accordance with our inclusion and exclusion criteria (Fig 1). Ultimately, 30 studies [10–17, 26–47] were eligible for this meta-analysis. Table 2 lists their general characteristics. There were 139,167,581 subjects in total, and most of the studies were from the Asia-Pacific region. The sample sizes of the 30 studies varied from 73 [39] to 138,877,499 participants [12], but the majority of studies (n = 25) enrolled >500 subjects. The participants’ average age ranged 33–61 years. Ten studies [36–41, 44–47] only reported MetS components in the form of continuous variables, and they mainly affected the pooled SMD of MetS components. Consequently, we did not consider in our analysis the MetS criteria they used. In other words, whether these studies [36–41, 44–47] meet the MetS criteria (Q4: The MetS and its components were defined accurately) did not affect the statistical results (SMD), so they were labeled with “UR” (unrelated) for Q4 in Table 3. The remaining 20 studies [10–17, 26–35, 42, 43] reported ORs or crude data for calculating the ORs. The MetS criteria used in these 20 studies was similar, but not identical. S1 Table lists the detailed criteria applied in these 20 studies.

**Table 2:** Characteristics of the studies included in the meta-analysis.

Author, year	Region	Study design	General population	Age^‡^	HBsAg (+) (male%)^£^	HBsAg (-) (male%)^£^
Huang CY, 2016 [10]	Taiwan	cross section	Yes	36.2±3.8 *vs*. 36.1±3.9	2982 (54.4)	14048 (41.4)
Katoonizadeh A, 2016 [15]	Iran	Unclear	Yes	56.1±8.3 *vs*. 56.0±8.0	2249 (52.4)	10532 (47.0)
Fan JY, 2015 [27]	Taiwan	cross section	Yes	49.8±16.4	1265 (50.1)	5540 (42.1)
Ha M, 2015 [11]	China	cross section	Patients	40±13 *vs*. 44±15	121 (54.5)	263 (56.3)
Hsu CS, 2015 [26]	Taiwan	cross section	Yes	51.8±9.6 *vs*. 51±12.9	187 (56.7)	184 (54.4)
Choi JS, 2015 [28]	Korea	cross section	Yes	47.1±15.1	209 (51.2)	4899 (41.6)
Park B, 2014 [29]	Korea	cross section	Yes	>30	916 (48.3)	23355
Jinjuvadia R, 2014 [12]	US	cross section	Yes	>18	593594 (68.1)	138283905 (47.5)
Jarčuška P, 2014 [16]	Slovakia	cross section	Yes	33.8±6.9 *vs*. 34.1 ± 8.4	66	771
Chung TH, 2014 [30]	Korea	cross section	Yes	45.7±5.7 *vs*. 50.0±6.0(m)^§^	521 (83.9)	8953 (80.0)
				45.4±9.4 *vs*. 47±9.9(f)		
Liu PT, 2013 [31]	Taiwan	cross section	Yes	47±11	1036 (64.1)	6659 (56.6)
Li WC, 2013 [32]	Taiwan	cross section	Yes	40.7±13.2	3408 (62.4)	22897 (54.2)
Wong VWS, 2012 [33]	Hong Kong	cross section	Yes	49±10 *vs*. 48±11	91	922
Hsu CS 2012 [34]	Taiwan	cross section	Patients	unclear	322 (53.1)	870 (53.7)
Chen JY, 2010 [35]	Taiwan	cross section	Yes	60.9±11.8	6133	50203
Ishizaka N, 2008 [17]	Japan	cross section	Yes	55.3±10.6 *vs*. 53.1±10.6	130 (71.5)	12333 (64.2)
Yang KC, 2007 [42]	Taiwan	cross section	Yes	48.0±9.6 *vs*. 48.4±10.7	87 (72.4)	421 (76.48)
Luo B, 2007 [13]	China	cross section	Yes	43.5 (32–87)	858 (75.8)	6579 (64.6)
Lin YC, 2007 [43]	Taiwan	cross section	Yes	45.9±8.8 *vs*. 46.3±9.5	817 (59.9)	4589 (49.5)
Jan CF, 2006 [14]	Taiwan	cross section	Yes	30–79	5994	41699
Chiang CH, 2013 [36]^†^	Taiwan	cross section	Yes	33.0±8.6 *vs*. 23.5±2.4	147 (76.9)	359 (63.0)
Cheng YL, 2013 [37] ^†^	Taiwan	cross section	Yes	49.5±11.5 *vs*. 52.2±13.3	3642 (59.3)	29797 (54.4)
Lee JG, 2012 [38]^†^	South Korea	cross section	Yes	48.9±10(m); 48.6±10(f) ^¶^	7880 (48.9)	
Karsen H, 2012 [39]^†^	Turkey	cross section	Unclear	36.2±14.2 *vs*. 35.2±14.1	34 (47.1)	39 (43.6)
Dai F, 2012 [40]^†^	China	cross section	Patients	38.7±9.5 *vs*. 37.2±10.6	68 (69.1)	67 (59.7)
Huang ZS, 2010 [41]^†^	Taiwan	cross section	Yes	52.7±0.7 *vs*. 55.1±0.3	143 (79.0)	1090 (72.5)
Wang CC, 2008 [47]^†^	Taiwan	cross section	Yes	44.6±1.4 *vs*. 46.8±0.4	50 (60)	457 (46.6)
Targher G, 2007 [45]^†^	Italy	cross section	Patients	47 ± 3 *vs*. 46 ± 3	35 (65.7)	60 (68.0)
Moritani M, 2005 [44]^†^	Japan	cross section	Yes	48.3±1.3 *vs*. 49.3±0.2	39 (89.7)	1736 (65.3)
Su TC, 2004 [46]^†^	Taiwan	cross section	Yes	40.4±7.5 *vs*. 41.1±8.3	195 (36.9)	1135 (29.3)

HBsAg, hepatitis B surface antigen.

^†^ These studies only reported components of MetS in the form of continuous variables.

^‡^ Age was usually expressed as “HBsAg-positive group” vs. “control group” or the overall age distribution including HBsAg-positive and control group.

^§^ “age of HBsAg-positive group” vs. “age of control group” in male subgroup (m) and female subgroup (f), respectively.

^¶^ Overall age distribution in male subgroup (m) and female subgroup (f), respectively.

^£^ Data in parentheses are the percentage of males.

**Table 3:** Methodological quality of eligible studies.

Author, year	Q1	Q2	Q3	Q4	Q5	Q6	Q7	Q8	Q9	Score
Huang CY, 2016 [10]	yes	yes	yes	yes	yes	yes	yes	yes	yes	9
Katoonizadeh A, 2016 [15]	yes	yes	yes	yes	yes	yes	yes	yes	yes	9
Fan JY, 2015 [27]	yes	yes	yes	no	yes	yes	no	no	no	5
Ha M, 2015 [11]	no	yes	yes	yes	yes	yes	yes	yes	yes	8
Hsu CS, 2015 [26]	yes	yes	yes	yes	yes	yes	yes	yes	no	8
Choi JS, 2015 [28]	yes	yes	yes	yes	yes	yes	UC	yes	yes	8
Park B, 2014 [29]	yes	yes	yes	yes	yes	yes	no	no	no	6
Jinjuvadia R, 2014 [12]	yes	yes	yes	yes	yes	yes	yes	yes	yes	9
Jarčuška P, 2014 [16]	yes	yes	yes	no	yes	yes	yes	yes	no	7
Chung TH, 2014 [30]	yes	yes	yes	yes	yes	yes	UC	yes	yes	8
Liu PT, 2013 [31]	yes	yes	yes	yes	yes	yes	yes	yes	yes	9
Li WC, 2013 [32]	yes	yes	yes	yes	yes	yes	no	no	no	6
Wong VWS, 2012 [33]	yes	yes	yes	yes	yes	yes	yes	yes	yes	9
Hsu CS, 2012 [34]	no	yes	yes	yes	yes	yes	yes	yes	no	7
Chen JY, 2010 [35]	yes	yes	yes	yes	yes	yes	no	no	no	6
Ishizaka N, 2008 [17]	yes	yes	yes	yes	yes	yes	yes	yes	no	8
Yang KC, 2007 [42]	yes	yes	yes	no	yes	yes	yes	no	no	6
Luo B, 2007 [13]	yes	yes	yes	no	yes	yes	no	yes	no	6
Lin YC, 2007 [43]	yes	yes	UC	yes	yes	yes	no	no	no	5
Jan CF, 2006 [14]	yes	yes	yes	no	yes	yes	no	yes	no	6
Chiang CH, 2013 [36]	yes	no	yes	UR	yes	yes	yes	no	yes	7
Cheng YL, 2013 [37]	yes	yes	yes	UR	yes	yes	yes	no	no	7
Lee JG, 2012 [38]	yes	yes	yes	UR	yes	yes	yes	no	no	7
Karsen H, 2012 [39]	UC	yes	yes	UR	yes	yes	yes	yes	no	7
Dai F, 2012 [40]	no	yes	yes	UR	yes	yes	yes	yes	no	7
Huang ZS, 2010 [41]	yes	yes	yes	UR	yes	yes	yes	no	no	7
Wang CC, 2008 [47]	yes	yes	yes	UR	yes	yes	yes	yes	no	8
Targher G, 2007 [45]	no	yes	yes	UR	yes	yes	yes	yes	no	7
Moritani M, 2005 [44]	yes	yes	yes	UR	yes	yes	yes	yes	yes	9
Su TC, 2004 [46]	yes	yes	yes	UR	yes	yes	no	no	no	6

UC: unclear;

UR: unrelated. The last 10 studies [36–41, 44–47] in the table reported only metabolic syndrome (MetS) components in the form of continuous variables, and they mainly affected the pooled standardized mean difference (SMD) of the MetS components. SMD was not related to the diagnostic criteria of MetS. Whether these studies [36–41, 44–47] meet Q4 (Q4: MetS and its components were defined accurately) did not affect the statistical results (SMD), so they were labeled “UR” for Q4. The first 20 studies in the table reported OR or crude data for calculating the OR, and the MetS criteria they used affected the statistical results (pooled ORs) directly. Therefore, these studies were carefully investigated to confirm whether they met Q4 (Q4: MetS and its components were defined accurately).

### Methodological quality assessment

Table 3 lists the methodological quality of the studies; the average score of all 30 studies was 7.23. Five studies [11, 34, 39, 40, 45] did not collect information on HBsAg-positive subjects from the general population, but from patients in the infection department. One study [36] enrolled university graduates as the healthy controls, who were much younger than the HBsAg-positive group. One study [43] did not define the HBsAg-positive group explicitly. The definition criteria of MetS differed slightly in these studies even though most of them were based on National Cholesterol Education Program Adult Treatment Expert Panel III (ATP III) [21] (S1 Table). Five studies involved the distinctive definition of MetS or its components. Jarčuška *et al*. [16] considered that MetS must present with central obesity. Increased BP was defined as systolic BP ≥ 140 mmHg or diastolic BP ≥ 90 mmHg in three studies [13, 27, 42] and as systolic BP ≥ 135 mmHg or diastolic BP ≥ 90 mmHg in one study [14]. The Q7, Q8, and Q9 checklists were mainly used to control confounders. Ten studies involved the confounding of HCV. Eleven studies did not control for confounding of age and sex well, while 20 studies did not control for confounding of lifestyle well.

### HBsAg positivity and MetS

**Fig 2:**
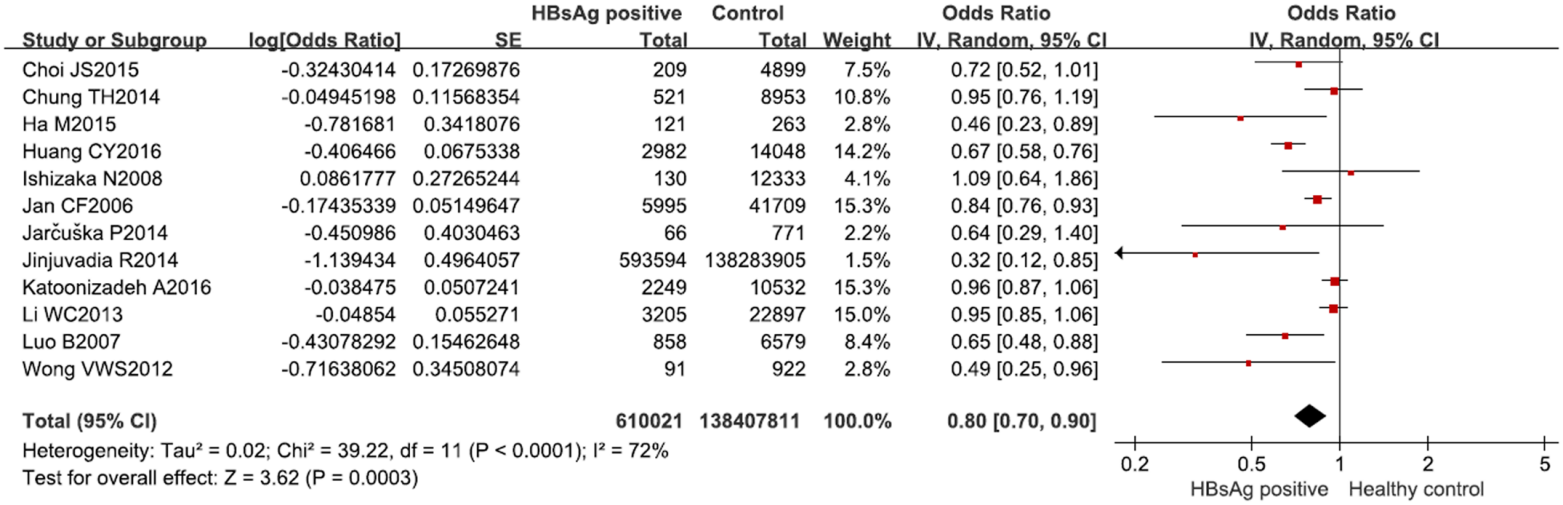
Forest plot of the prevalence of MetS in HBsAg-positive subjects versus healthy controls.

Twelve studies [10–17, 28, 30, 32, 33] reported the OR for HBsAg positivity and prevalence of MetS. In all, 610,021 HBsAg-positive subjects and 138,407,811 healthy controls were enrolled in the meta-analysis. The pooled OR for HBsAg positivity and MetS prevalence was 0.80 (95% CI, 0.70–0.90, I^2^ = 72%, *P* < 0.01) (Fig 2), indicating an inverse association between HBsAg positivity and MetS prevalence. Table 4 lists the subgroup analysis results. The inverse relationship was robust in all but the female subgroup. In the general population, the pooled OR from 11 studies [10, 12–17, 28, 30, 32, 33] was 0.81 (95% CI, 0.72–0.92, I^2^ = 72%, *P* < 0.01). The pooled OR from nine studies [10–12, 15, 17, 28, 30, 32, 33] that rigorously defined MetS with ATP III was 0.80 (95% CI, 0.68–0.94, I^2^ = 77%, *P* < 0.01). After excluding the confounder of HCV, the pooled OR was 0.70 (95% CI, 0.53–0.91, I^2^ = 80%, *P* < 0.01). The pooled OR from data adjusted for confounders was 0.73 (95% CI, 0.61–0.88, I^2^ = 63%, *P* = 0.02). This inverse association was also found in the male subgroup (OR = 0.85; 95% CI, 0.74–0.98; I^2^ = 64%, *P* = 0.01), but not in the female subgroup (OR = 0.91; 95% CI, 0.74–1.11; I^2^ = 66%, *P* = 0.008). Furthermore, the heterogeneity did not decrease through subgroup analysis, therefore the specific factor leading to heterogeneity was not found.

**Table 4:** Results of subgroup analysis according to quality assessments.

Groups^†^	MetS	Elevated WC	Elevated TG	Reduced HDL-C	Elevated BP	Elevated FBG
All	0.80 (0.70–0.90)^‡^; I^2^ = 72%, *P*<0.01; n = 12	0.97 (0.91–1.04); I^2^ = 50%, *P* = 0.03; n = 11	0.62 (0.59–0.64); I^2^ = 0%, P = 0.52; n = 14^§^	0.98 (0.83–1.14); I^2^ = 85%, *P*<0.01; n = 13^§^	1.00 (0.80–1.25); I^2^ = 95%, *P*<0.01; n = 11^§^	0.94 (0.90–0.98); I^2^ = 21%, *P* = 0.23; n = 13^§^
Male	0.85 (0.74–0.98); I^2^ = 64%, *P* = 0.01; n = 6	0.91 (0.81–1.02); I^2^ = 51%, *P* = 0.11; n = 4	--	1.21 (1.05–1.40); I^2^ = 50%, *P* = 0.11; n = 4	0.97 (0.80–1.17); I^2^ = 5%, *P* = 0.35; n = 3	0.63 (0.39–1.00); I^2^ = 89%, *P*<0.01; n = 4
Female	0.91 (0.74–1.11); I^2^ = 66%, *P* = 0.008; n = 6	0.95 (0.84–1.09); I^2^ = 0%, *P* = 0.41; n = 4	--	0.82 (0.50–1.35); I^2^ = 82%, *P* = 0.009; n = 4	0.95 (0.66–1.39); I^2^ = 0, *P* = 0.89; n = 3	1.00 (0.88–1.14); I^2^ = 0, *P* = 0.80; n = 4
Q1 (general population)	0.81 (0.72–0.92); I^2^ = 72%, *P*<0.01; n = 11	0.97 (0.91–1.04); I^2^ = 55%, *P* = 0.02; n = 10	--	0.95 (0.83–1.09); I^2^ = 78%, *P*<0.01; n = 11	0.91 (0.87–0.96); I^2^ = 0, *P* = 0.63; n = 9	0.94 (0.90–0.99); I^2^ = 27%, *P* = 0.18; n = 11
Q4 (accurate diagnosis)	0.80 (0.68–0.94); I^2^ = 77%, *P*<0.01; n = 9	0.99 (0.94–1.05); I^2^ = 0%, *P* = 0.93; n = 6	--	0.98 (0.82–1.16); I^2^ = 86%, *P*<0.01; n = 12	0.95 (0.88–1.02); I^2^ = 0, *P* = 0.63; n = 7	0.93 (0.87–0.99); I^2^ = 1%, *P* = 0.42; n = 7
Q7 (included and excluded criterion)	0.70 (0.53–0.91); I^2^ = 80%, *P*<0.01; n = 7	0.93 (0.83–1.04); I^2^ = 61%, *P* = 0.02; n = 7	--	0.94 (0.72–1.21); I^2^ = 89%, *P*<0.01; n = 9	0.92 (0.85–1.00); I^2^ = 0, *P* = 0.68; n = 6	0.96 (0.91–1.03); I^2^ = 37%, *P* = 0.14; n = 7
Q8 and Q9 (control confounding factors)	0.73(0.61–0.88); I^2^ = 63%, *P* = 0.02; n = 6	0.99 (0.91–1.08); I^2^ = 64%, *P* = 0.04; n = 4	--	0.88 (0.83–0.94); I^2^ = 0%, *P* = 0.47; n = 6	0.90 (0.85–0.94); I^2^ = 0, *P* = 0.69; n = 4	0.97 (0.90–1.03); I^2^ = 57%, *P* = 0.08; n = 4

MetS, metabolic syndrome; WC, waist circumference; TG, triglycerides; HDL-C, high-density lipoprotein cholesterol; BP, blood pressure; FBG, fasting blood glucose.

^†^ Grouped according to checklist of quality assessment (Tables 1 and 3).

^‡^ The data in each grid are the OR (95% CI of OR); the parameters of heterogeneity (I^2^, *P*-value); the number of included studies.

^§^ The studies included for calculating the pooled OR here were not identical to those for calculating the pooled SMD.

For “Elevated TG”, the pooled OR was from 14 studies [10–16, 26, 28, 30, 31, 33–35], and the SMD was from 14 studies [10, 16, 17, 26, 31, 34, 36, 37, 39, 42, 44–47]. They are not identical.

Similarly, for “Reduced HDL-C”, the pooled OR was from 13 studies [10–16, 26, 28, 30, 31, 33, 34], and the SMD was from 19 studies [10, 15–17, 26, 28, 30–34, 37–39, 42, 44–47].

For “Elevated BP”, the pooled OR was from 11 studies [10–15, 28, 30, 31, 33, 42]; the SMD of systolic BP was from 10 studies [10, 11, 17, 31, 33, 36, 37, 42, 44, 45], and the SMD of diastolic BP was from nine studies [10, 11, 17, 31, 33, 36, 37, 42, 45].

For “Elevated FBG”, the pooled OR was from 13 studies [10–15, 27–31, 33, 34], and the SMD was from 16 studies [10, 11, 16, 17, 26, 31, 33, 36, 37, 40–42, 44–47].

### HBsAg positivity and central obesity

WC and BMI are two common indices for assessing central obesity. Eleven studies [10–12, 14–16, 26, 28, 30, 33, 43] involving 606,706 HBsAg-positive subjects and 138,369,865 healthy controls reported the OR for HBsAg positivity and increased WC. The pooled OR was 0.97 (95% CI, 0.91–1.04; I^2^ = 50%, *P* = 0.03) (S1 Fig), indicating that HBsAg positivity was neither a risk factor nor a protective factor for increased WC, and further subgroup analysis grouped according to quality assessment confirmed this. The pooled OR from six studies [10, 11, 14, 26, 33, 43] that defined central obesity as WC > 90 cm in men or >80 cm in women was 0.99 (95% CI, 0.94–1.05; I^2^ = 0%, *P* = 0.93). The heterogeneity also decreased in subgroups stratified by sex; the conclusion was identical to the total pooled OR (Table 4). Additionally, six studies [13, 16, 26, 27, 34, 43] reported the OR of BMI, and the pooled OR was 0.99 (95% CI, 0.95–1.04; I^2^ = 0%, *P* = 0.65), which was consistent with WC.

### HBsAg positivity and elevated TG

**Fig 3:**
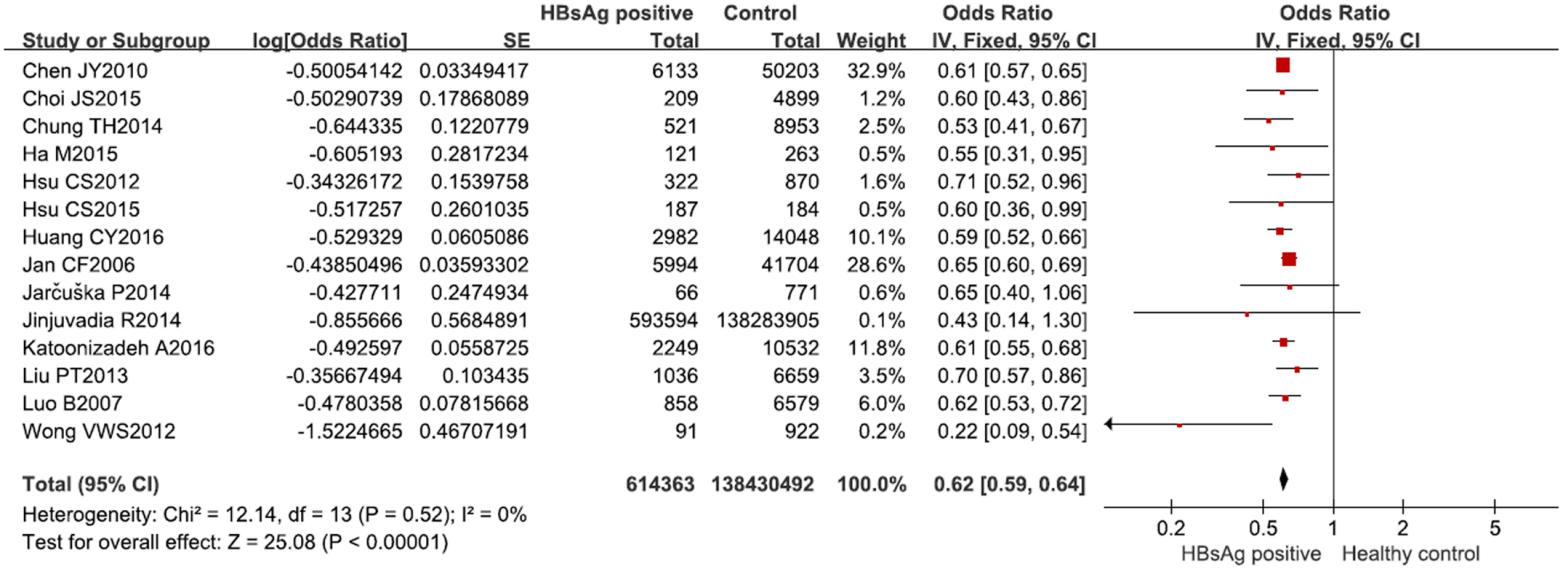
Forest plot of the prevalence of elevated TG in HBsAg-positive subjects versus healthy controls.

Fourteen studies [10–16, 26, 28, 30, 31, 33–35] involving 614,363 HBsAg-positive subjects and 138,430,492 healthy controls reported the OR for HBsAg positivity and increased circulating TG levels. The total OR of these 14 studies was 0.62 (95% CI, 0.59–0.64; I^2^ = 0%, *P* = 0.52) (Fig 3), indicating that HBsAg positivity is inversely associated with elevated TG. The heterogeneity among the included studies was so low that the subsequent subgroup analysis was omitted. The SMD of the 14 studies [10, 16, 17, 26, 31, 34, 36, 37, 39, 42, 44–47] was -0.39 (95% CI, -0.59 to -0.18; I^2^ = 98%, *P* < 0.001), indicating that the HBsAg-positive subjects had lower TG than the healthy controls. Although the OR and SMD were calculated from different studies, they revealed a consistent trend.

### HBsAg positivity and reduced HDL-C

Thirteen studies [10–16, 26, 28, 30, 31, 33, 34] involving 605,924 HBsAg-positive subjects and 138,363,354 healthy controls reported the OR for HBsAg positivity and reduced HDL-C. The total OR of the 13 studies was 0.98 (95% CI, 0.83–1.14, I^2^ = 85%, *P* < 0.01) (see S2 Fig), indicating that HBsAg positivity was not associated with reduced HDL-C. However, the pooled OR of six studies [10, 12–14, 16, 31] that controlled the confounding factors revealed an inverse relationship between HBsAg positivity and reduced HDL-C (OR = 0.88; 95% CI, 0.83–0.94; I^2^ = 0%, *P* = 0.47). The dramatic decrease in heterogeneity was due to adjusting for confounding factors (age, sex at least). However, the dramatic decrease in heterogeneity rendered the results more reliable, the inverse relationship was still weak.

### HBsAg positivity and elevated BP

Eleven studies [10–15, 28, 30, 31, 33, 42] reported the OR for HBsAg positivity and elevated BP, and only two [14, 15] reported that HBsAg positivity was associated with increased BP. The pooled OR of all 11 studies was 1.00 (95% CI, 0.80–1.25; I^2^ = 95%, *P* < 0.001) (see S3 Fig). After excluding the two studies [14, 15], the heterogeneity decreased significantly, and the combined OR from the remaining nine studies [10–13, 28, 30, 31, 33, 42] was 0.94 (95% CI, 0.88–1.01, I^2^ = 0%, *P* = 0.76). The subgroup that included seven studies [10–12, 28, 30, 31, 33] based on ATP III (systolic BP ≥ 130 mmHg or diastolic BP ≥ 85 mmHg) also showed no relationship between HBsAg positivity and increased BP (OR = 0.95; 95% CI, 0.88–1.02; I^2^ = 0%, *P* = 0.63). Additionally, similar trends were found in the SMD of systolic BP and diastolic BP. In conclusion, HBsAg positivity was neither a risk factor nor a protective factor for increased BP, and the difference in BP between HBsAg-positive subjects and healthy controls was not significant.

### HBsAg positivity and elevated FBG

Thirteen studies [10–15, 27–31, 33, 34] reported the OR for HBsAg positivity and elevated FBG. The total OR of these 13 studies, which involved 610,127 HBsAg-positive subjects and 138,408,194 controls, was 0.94 (95% CI, 0.90–0.98; I^2^ = 21%, *P* = 0.23) (see S4 Fig), indicating that HBsAg positivity is inversely associated with increased FBG, but this inverse relationship was not robust in the subsequent subgroup analysis (Table 4). Seven studies [11, 15, 29–31, 33, 34] defined elevated FBG as ≥100 mg/dL, and the pooled OR was 0.93 (95% CI, 0.87–0.99; I^2^ = 1%, *P* = 0.42). Six studies [10, 12–14, 27, 28] defined elevated FBG as ≥110 mg/dL, and the pooled OR was 0.95 (95% CI, 0.89–1.01; I^2^ = 45%, *P* = 0.11). The SMD derived from 16 studies [10, 11, 16, 17, 26, 31, 33, 36, 37, 40–42, 44–47] was 0.03 (95% CI, -0.21 to 0.27; I^2^ = 99%, *P* < 0.0001). Overall, the effect of HBsAg positivity on glucose homeostasis appeared slight. However, further research is required to confirm this.

### Publication bias

Publication bias was not detected by Egger’s test or Begg’s test (Table 5). For Egger’s test, the publication bias 95% CI of each group included zero and *P* > 0.05, so there was no statistical difference between publication bias and zero, meaning no publication bias was present; Begg’s test derived the same conclusion. Taken together, this indicates that there was no publication bias in our meta-analysis.

**Table 5:** Analysis of publication bias of the included studies.

Group	Studies	Begg’s test (*P*-value)	Egger’s test	
			*P*-value	95% CI of bias
MetS	12	0.086	0.089	-3.34 to 0.28
Elevated BMI	6	0.707	0.300	-0.88 to 2.21
Elevated WC	11	0.119	0.506	-2.03 to 1.08
Elevated TG	14	0.274	0.228	-1.37 to 0. 36
Reduced HDL-C	13	0.583	0.866	-3.01 to 2.57
Elevated BP	11	1.000	0.902	-5.66 to 5.06
Elevated FBG	13	0.161	0.123	-2.09 to 0.29

MetS, metabolic syndrome; BMI, body mass index; WC, waist circumference; TG, triglycerides; HDL-C, high-density lipoprotein cholesterol; BP, blood pressure; FBG, fasting blood glucose.

## Discussion

In this meta-analysis, HBsAg-positive individuals had lower prevalence of MetS. This negative association remained robust after adjustment for confounding factors (e.g., age, sex). Meanwhile, a strong inverse relationship was demonstrated between HBsAg positivity and elevated TG (one component of MetS). There was a slight effect of HBsAg positivity on glucose homeostasis. The total OR of all eligible studies indicated no association between HBsAg positivity and reduced HDL-C, but OR controlled for the confounding factors revealed a slight inverse relationship. Additionally, it was confirmed that HBsAg positivity is not associated with central obesity and increased BP. Overall, we speculate that HBsAg positivity protects against the incidence of MetS mainly due to its negative effect on elevated TG. Naturally, further research is required to confirm this.

There was a negative association between HBsAg positivity and the prevalence of MetS, and HBsAg positivity is closely related to HBV. HBV may prevent the occurrence of MetS instead of promoting it. That is, HBV may protect humans against MetS. HBV is considered a “metabolovirus”, as it adopts a regulatory system that is unique to the major hepatic metabolic genes that control hepatic glucose and lipid metabolism [48]. HBV infection alters bile acid and cholesterol metabolism as a consequence of impaired bile acid uptake [48]. Besides, HBV X protein induces the transcriptional activation of peroxisome proliferator–activated receptor γ (PPARγ) [49]. The activation of PPARγ gene expression during HBV replication boosts the increase in circulating adiponectin levels [50, 51]. Adiponectin has anti-inflammatory effects and protects against insulin resistance. It is inversely associated with BMI, type 2 diabetes mellitus, and several metabolic disorders [51, 52]. Additionally, nonalcoholic steatohepatitis is considered the hepatic manifestation of MetS. A meta-analysis and several large-cohort studies have proven that HBV has a protective effect against the development of hepatic steatosis [6, 53]. The evidence described above all support the inverse relationship between HBsAg positivity and the prevalence of MetS; however, prospective studies are warranted to elucidate the exact mechanism and to validate the inverse relationship.

A recent review [6] has also shown an inverse relationship between HBV and increased TG. The liver is the main organ for lipid metabolism, and hepatic dysfunction such as inflammation, liver fibrosis, cirrhosis, and hepatocellular carcinoma may occur during HBV infection. These processes all influence lipid biosynthesis and metabolism and relate to the change in TG levels [35]. Kim *et al*. [49] reported that HBV X protein inhibits the secretion of apolipoprotein B. Apolipoprotein B in the liver is an important glycoprotein for the transport of TG-rich very low–density lipoprotein cholesterol and low-density lipoprotein cholesterol. Therefore, HBV X protein increases rapidly upon the active replication of HBV. Then, it inhibits very low–density lipoprotein cholesterol and low-density lipoprotein cholesterol production and promotes TG accumulation in hepatocytes, decreasing TG in the blood. Additionally, increased levels of adiponectin caused by HBV replication reduce serum TG levels and increase HDL-C levels [54]. Besides TG, accumulating evidence has revealed that chronic HBV infection is also inversely associated with other lipid profiles, including cholesterol and low-density lipoprotein cholesterol [6], and we found a similar trend. In our study, the OR for increased cholesterol from four studies [16, 31, 34, 35] was 0.76 (95%CI, 0.65–0.89), and the SMD from 13 studies [10, 11, 16, 17, 31, 33, 36, 37, 40, 42, 44, 46, 47] was -1.24 (95%CI, -1.64 to -0.84). The SMD of low-density lipoprotein cholesterol from 10 studies [16, 31, 33, 37–39, 42, 45–47] was -0.43 (95%CI, -0.69 to -0.16). The pooled OR of six studies that controlled the confounding factors revealed a slight inverse relationship between HBsAg positivity and reduced HDL-C. In fact, there was interaction between HBV infection and lipid metabolism. Moderate-severe hepatic steatosis may contribute to HBsAg seroclearance due to steatosis-induced apoptosis and inflammation [55]. In short, the possible mechanism for HBsAg positivity with lower TG levels could be related to viral factors and host factors. Furthermore, the weak inverse relationship between HBsAg positivity and reduced HDL-C should be confirmed via further investigation.

The inverse relationship between HBsAg positivity and increased FBG was statistically significant, but was weak in the clinic. The relationship between HBV and insulin resistance remains inconclusive and awaits further studies for clarification [6]. However, it is worth pointing out that cirrhosis and poor glycemic control are closely associated [56, 57]. It has been speculated that peripheral insulin clearance is reduced because of cirrhosis, and then insulin resistance and glucose abnormalities occur secondary to hyperinsulinemia [58].

To the best of our knowledge, this is the first meta-analysis to investigate the relationship between HBsAg positivity and MetS (including its components). Additionally, this meta-analysis was performed rigorously according to a proposal for reporting meta-analysis of observational studies [18]. Although Wang *et al*. [6] also focused on the association between HBV infection and MetS, theirs was more of an excellent review than a meta-analysis. Second, most of the included studies enrolled >500 subjects, and the large sample size made the conclusion more credible.

There are several limitations to the present meta-analysis. First, the majority of eligible studies were cross-sectional studies, which always demonstrate the least evidence among the three types of observational studies (case-control, cohort, cross-sectional). Additionally, time is an important factor that should be considered, as HBsAg-positive individuals may have different outcomes. Unfortunately, it was difficult to assess the impact of time in this meta-analysis, which we attribute to the cross-sectional nature of the included studies. Second, because only HBsAg was tested and/or it was tested for only once in most of the eligible studies, various conditions related to HBsAg were not taken into account. An HBsAg-positive individual may be a healthy carrier, a patient with chronic active hepatitis, or a patient with liver cirrhosis. Although most studies focused on the general population and most HBsAg-positive subjects may be HBV carriers in this meta-analysis, further stratification of HBsAg status is still needed to assess the exact role of HBsAg in the development of MetS in the future. Third, both age and gender play an important role in the natural history of chronic HBV infection. Unfortunately, the studies included in the subgroup analysis based on these two factors were very limited; however, the negative association between HBsAg passivity and MetS remained robust after adjustment for confounding factors (e.g., age, sex). Fourth, with respect to the definition of MetS, we were not concerned whether drug treatment was an alternate indicator. Finally, we were unsuccessful in obtaining supplemental information from several authors; however, no publication bias was detected.

Our meta-analysis has several implications for future research. First, a prospective large-cohort study is needed to validate our conclusion. In this regard, the Newcastle–Ottawa Scale [19] describes the requirements for a rigorous study design and methodology and is a good tool for guiding study design. The unified definition of MetS [4] should be used. As described above, some important factors, such as time, age, gender, and various conditions related to HBsAg, should be taken into account thoroughly in future research. On the other hand, the physiopathological mechanism of the inverse association between HBsAg positivity and MetS requires further research.

In conclusion, serum HBsAg positivity is inversely associated with MetS. Among the five components of MetS, elevated triglycerides had the strongest inverse relationship with HBsAg positivity.

## Supporting information

S1 Checklist PRISMA 2009 checklist. (DOC)

S1 Fig Meta-analysis of the prevalence of elevated WC in HBsAg positivity versus healthy control (forest plot). (TIF)

S2 Fig Meta-analysis of the prevalence of reduced HDL-C in HBsAg positivity versus healthy control (forest plot). (TIF)

S3 Fig Meta-analysis of the prevalence of elevated BP in HBsAg positivity versus healthy control (forest plot). (TIF)

S4 Fig Meta-analysis of the prevalence of elevated FBG in HBsAg positivity versus healthy control (forest plot). (TIF)

S1 Table Diagnostic criteria of MetS and its components in the included studies. (DOC)

S1 Text The electronic search strategy for PubMed database. (DOCX)

## Abbreviations

ATP III: National Cholesterol Education Program Adult Treatment Expert Panel III
BMI: body mass index
BP: blood pressure
Cl: confidence interval
FBG: fasting blood glucose
HBsAg: hepatitis B virus surface antigen
HBV: hepatitis B virus
HCV: hepatitis C virus
HDL-C: high-density lipoprotein cholesterol
MetS: metabolic syndrome
OR: odds ratio
PPARγ: peroxisome proliferator–activated receptor γ
SD: standard deviation
SMD: standardized mean difference
TG: triglyceride
WC: waist circumference
