# Supplementary figures and images for: Serum HBV surface antigen positivity is associated with low prevalence of metabolic syndrome: A meta-analysis
Source: PLoS One. 2017 May 15;12(5):e0177713. doi: 10.1371/journal.pone.0177713 (PMC5432182; doi:10.1371/journal.pone.0177713)

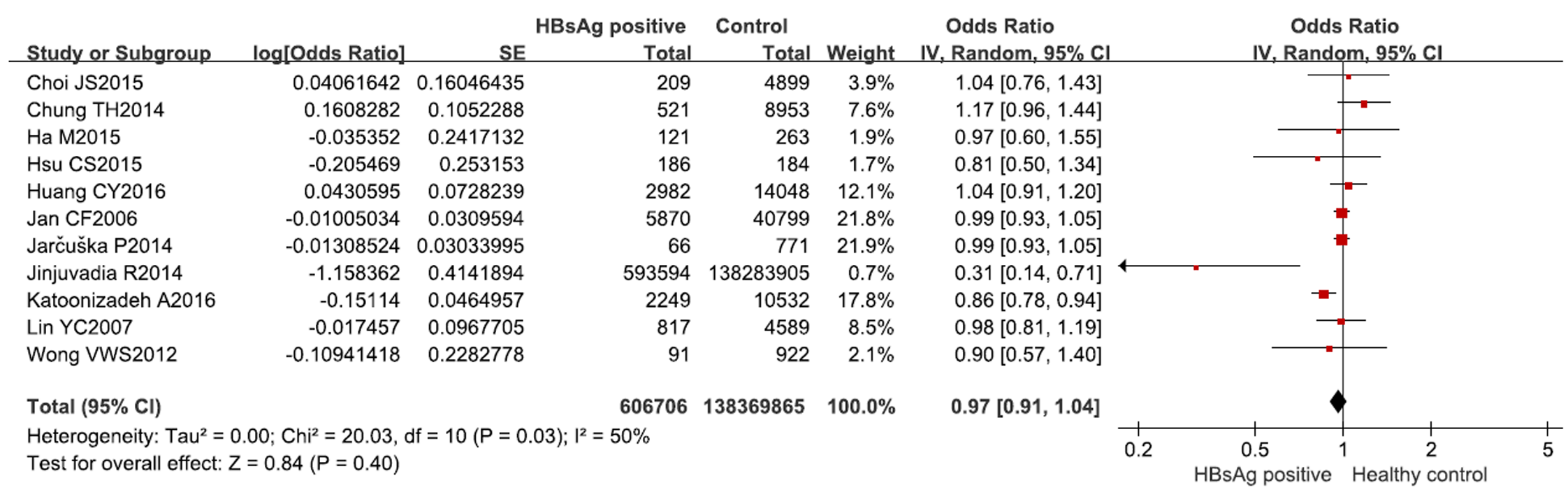

Supplement: S1 Fig — (TIF) [file pone.0177713.s002.tif]

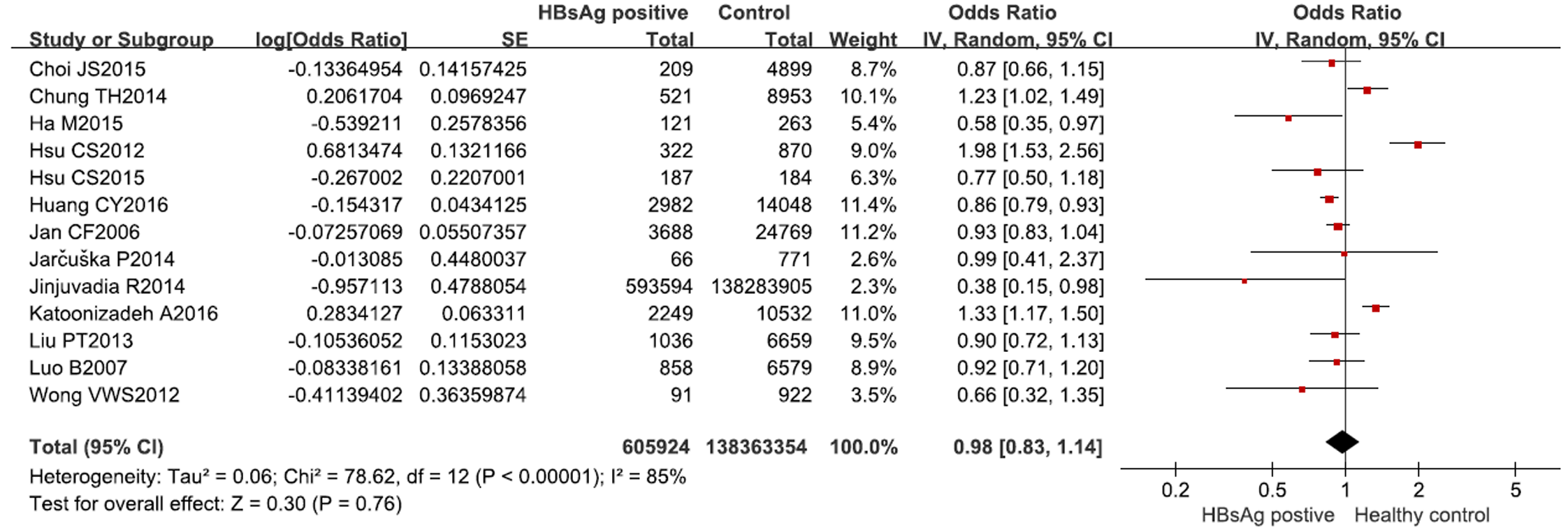

Supplement: S2 Fig — (TIF) [file pone.0177713.s003.tif]

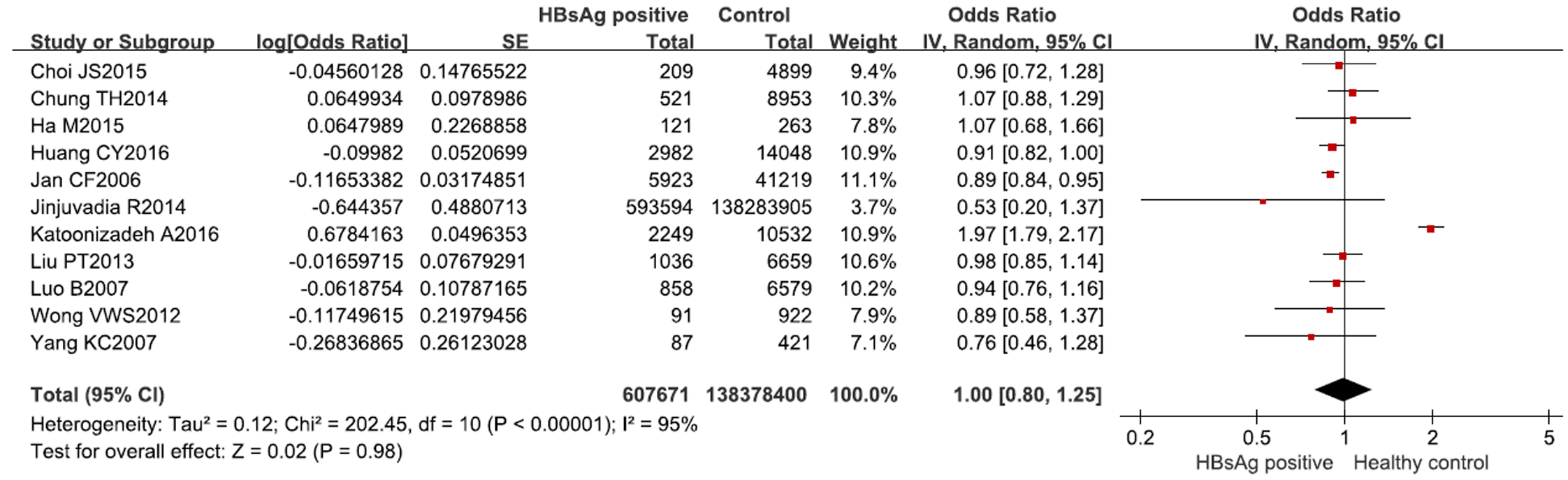

Supplement: S3 Fig — (TIF) [file pone.0177713.s004.tif]

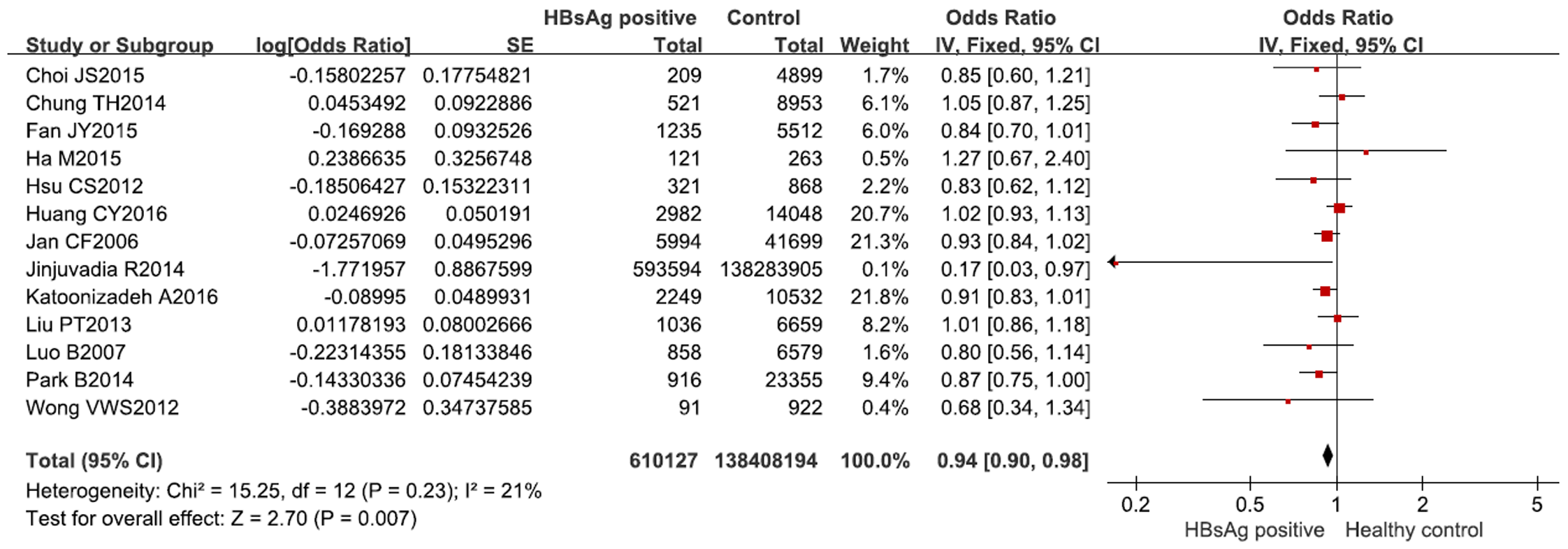

Supplement: S4 Fig — (TIF) [file pone.0177713.s005.tif]
