# Supplementary material for: Serum HBV surface antigen positivity is associated with low prevalence of metabolic syndrome: A meta-analysis
Source: PLoS One. 2017 May 15;12(5):e0177713. doi: 10.1371/journal.pone.0177713 (PMC5432182; doi:10.1371/journal.pone.0177713)
Supplement: S1 Table — (DOC) [file pone.0177713.s006.DOC]

**Serum** **HBV surface antigen positivity is associated with low prevalence of metabolic syndrome: A meta-analysis**

Yuanyuan Li, Ying Zhao, Jianping Wu^*^

**S1 Table** Diagnostic [criteria](javascript:void(0);) of MetS and its components in the included studies

| **Study ID** | **MetS** | **WC**  **(cm)** | **TG**  **(mg/dL)** | **HDL-C**  **(mg/dL)** | **BP**  **(mmHg)** | **FBG**  **(mg/dL)** | **Control of confounding factors** |
| --- | --- | --- | --- | --- | --- | --- | --- |
| Katoonizadeh A,2016 | ATPIII | M>95;F>95 | ≥150 | M<40; F<50 | >130/85 | ≥100 | Data were stratified by gender and adjusted for age, PLT, alcohol intake, smoking, exercise, and socioeconomic status in male and female group. |
| Huang CY,2016 | ATPIII | M≥90;F≥80 | ≥150 | M<40; F<50 | ≥130/85 | ≥110 | It was adjusted for age, gender, smoking status, alcohol consumption, and physical activity, BMI and ALT. |
| Fan JY, 2015 | -- | BMI>24kg/m^2^ | -- | -- | ≥140/90 | ≥110 | Not adjusted |
| Hsu CS,2015 | -- | M>90;F>80  BMI≥25kg/m^2^ | ≥150 | M<40; F<50 | -- | -- | There was no significantly difference in age, sex and BMI. |
| Ha M, 2015 | ATPIII | M>90;F>80 | >150 | M<40; F<50 | >130/85 | ≥100 | There was no significantly difference in age, male gender, prevalence of current smoking and drinking. |
| Choi JS,2015 | ATPIII | M>102;F>88 | >150 | M<40; F<50 | >130/85 | ≥110 | Total OR which was from all the participants was not adjusted.  Data were stratified by gender, and adjusted for age, location, smoking habits, alcohol consumption, exercise habits, income status, and education levels. |
| Park B,2014 | -- | -- | -- | -- | -- | >100 | Not adjusted. |
| Jinjuvadia R, 2014 | ATPIII | M>102;F>88 | >150 | M<40; F<50 | >130/85 | ≥110 | Adjusted for age, sex, race, smoking and alcohol status. |
| Jarčuška P, 2014 | Not ATPIII | M>94;F>80  BMI>30kg/m^2^ | >150 | M<40; F<50 | >130/85 | >100 | OR of TG, HDL-C and MetS were adjusted for age, sex, BMI and WC. OR of BMI and WC were adjusted only for age and sex. |
| Chung TH, 2014 | ATPIII | M>90;F>85 | ≥150 | M<40; F<50 | >130/85 | ≥100 | Total OR which was from all the participants was not adjusted.  Data were stratified by gender and adjusted for age, BMI, ALT, alcohol intake, smoking, exercise, family income, and educational status. |
| Liu PT,2013 | -- | -- | ≥150 | M<40; F<50 | >130/85 | ≥100 | OR of raised TG and reduced HDL-C were adjusted for age, sex, smoking status, alcohol consumption, BMI, and ALT. OR of raised BP and raised FBG were not adjusted. |
| Li WC,2013 | ATPIII | M>102;F>88 | >150 | M<40; F<50 | >130/85 | ≥100 | Not adjusted |
| Wong VWS, 2012 | ATPIII | M>90; F>80 | >150 | M<40; F<50 | ≥130/85 | ≥100 | Not adjusted. There was no significantly difference in age, sex, alcohol, and smoking. |
| Hsu CS,2012 | -- | M>90; F>80 BMI≥25kg/m^2^ | ≥150 | M<40; F<50 | -- | ≥100 | Not adjusted. |
| Chen JY,2010 | -- | BMI≥25kg/m^2^ | >150 | -- | -- | -- | Not adjusted. |
| Ishizaka N, 2008 | ATPIII | BMI≥25kg/m^2^ | ≥150 | M<40; F<50 | ≥130/85 | ≥110 | It was adjusted for age and sex. |
| Yang KC,2007 | -- | -- | -- | -- | ≥140/90 | -- | Not adjusted. |
| Luo B,2007 | Not ATPIII | BMI≥25kg/m^2^ | ≥150 | M<35; F<39 | ≥140/90 | ≥110 | It was adjusted for age and sex. |
| Lin YC,2007 | -- | M>90;F>80  BMI>24 kg/m^2^ | -- | -- | -- | -- | Not adjusted. |
| Jan CF,2006 | Not ATPIII | M>90;F>80 | ≥150 | M<40; F<50 | ≥135/85 | ≥110 | It was adjusted for age and sex. |

MetS, metabolic syndrome; ATPIII, National Cholesterol Education Program Adult Treatment Expert Panel III; WC, waist circumference; M, male; F, female; BMI, body mass index; TG, triglyceride; HDL-C, high-density lipoprotein cholesterol; BP, blood pressure; FBG, fasting blood glucose; ALT, alanine aminotransferase.
