# Supplementary material for: Serum HBV surface antigen positivity is associated with low prevalence of metabolic syndrome: A meta-analysis
Source: PLoS One. 2017 May 15;12(5):e0177713. doi: 10.1371/journal.pone.0177713 (PMC5432182; doi:10.1371/journal.pone.0177713)
Supplement: S1 Text — (DOCX) [file pone.0177713.s007.docx]

Search ((((((((((blood pressure[Title/Abstract]) OR hypertension[Title/Abstract])) OR ((("fasting blood glucose"[Title/Abstract]) OR "hyperglycaemia"[Title/Abstract]) OR "hyperglycemia"[Title/Abstract])) OR (("hdl"[Title/Abstract]) OR "high density lipoprotein"[Title/Abstract])) OR ((("triglyceride"[Title/Abstract]) OR "hypertriglyceridemia"[Title/Abstract]) OR "hyperlipemia")) OR ((obesity[Title/Abstract]) OR overweight[Title/Abstract])) OR ((((((("metabolic syndrome"[Title/Abstract]) OR "ms"[Title/Abstract]) OR "mets"[Title/Abstract]) OR "dysmetabolic syndrome"[Title/Abstract]) OR "insulin resistance"[Title/Abstract])) OR "Metabolic Syndrome X"[Mesh]))) AND ((((("hepatitis b"[Title/Abstract]) OR "hbv"[Title/Abstract]) OR "chb"[Title/Abstract])) OR "Hepatitis B"[Mesh]))
